# Update in Pathogenesis and Prospective in Treatment of Necrotizing Enterocolitis

**DOI:** 10.1155/2014/543765

**Published:** 2014-07-17

**Authors:** Gianluca Terrin, Antonella Scipione, Mario De Curtis

**Affiliations:** ^1^Department of Perinatal Medicine, Sapienza University, Viale del Policlinico 155, 00161 Rome, Italy; ^2^Department of Pediatrics and Infant's Neuropsychiatry, University of Rome “La Sapienza”, Rome, Italy

## Abstract

Necrotizing enterocolitis (NEC) is among the most common and devastating diseases in neonates and, despite the significant advances in neonatal clinical and basic science investigations, its etiology is largely understood, specific treatment strategies are lacking, and morbidity and mortality remain high. Improvements in the understanding of pathogenesis of NEC may have therapeutic consequences. Pharmacologic inhibition of toll-like receptor signaling, the use of novel nutritional strategies, and microflora modulation may represent novel promising approaches to the prevention and treatment of NEC. This review, starting from the recent acquisitions in the pathogenic mechanisms of NEC, focuses on current and possible therapeutic perspectives.

## 1. Introduction

Necrotizing enterocolitis (NEC) is an inflammatory disease of the intestine, often associated with sepsis and frequently complicated by perforation, peritonitis, and death. Despite the significant advances in neonatal clinical and basic science investigation, NEC often is an incurable disease. Specific therapeutic strategies are lacking because unknown etiology. Mortality rate is high and long-term prognosis in survivals is very poor. The inflammatory process, starting from intestinal mucosa, involves distant organs including the central nervous system, with an increased risk for neurodevelopment delay [1, 2]. The total annual estimated cost of caring for affected infants with NEC only in the United States ranges between $500 million and $1 billion [3]. For these reasons, NEC has become a priority for research.

The term “*necrotizing enterocolitis*” (NEC) often reflects a spectrum of intestinal conditions that differ with respect to the pathogenesis [1]. Classical form of NEC usually occurs in preterm neonates in the first 2 weeks of life. Spontaneous intestinal perforation, often diagnosed as NEC, occurs in a term neonates and it could be observed also several days after birth. This condition probably represents a different disease with a different pathogenesis, it is independent by modalities of feeding, and it is characterized by only minimal intestinal inflammation and/or necrosis [3, 4]. Our review focuses on recent advances in pathogenesis and potential therapeutic options of classical form of NEC.

## 2. Pathogenesis of Necrotizing Enterocolitis

**Figure 1:**
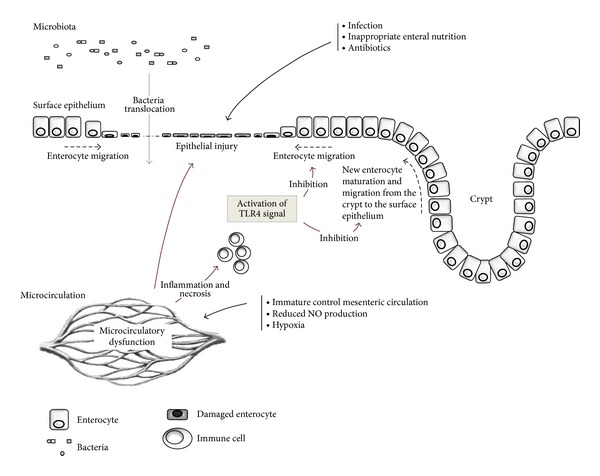
The role of TLR4 in epithelium injury and repair mechanisms. Many factors typical of prenatal birth such as infections, inappropriate enteral nutrition, antibiotics use, microcirculatory dysfunction, and hypoxia induce epithelial injury. Hyperactivation of TLR4 signaling affects the healing process favoring pathological bacteria translocation across epithelial barrier.

Despite the fact that the pathogenesis of NEC is considered multifactorial, recently the role of epithelium has emerged as central in the development of NEC. The loss of epithelial barrier allows pathogens translocation from the intestinal lumen to the mucosa (Figure 1). Innate immunity regulates epithelial barrier in experimental model and human cases of NEC.

### 2.1. Epithelial Barrier

The intestinal mucosa of the premature infant presents a persistent equilibrium state between injury and repair. Injury to the intestinal mucosa may depend on a variety of conditions typical of prematurity, including hypoxia [5, 6], infection [7], and starvation [8]. Microcirculatory dysfunction contributes to epithelial damage [9]. In physiological conditions, healing of epithelium begins immediately after the injury with mature enterocyte migration from health to the wounded area [10]. Subsequently, the proliferation of new enterocytes within the crypts of Lieberkuhn completes the process of repair [11]. It has been recently suggested that NEC is associated with a marked inhibition in both enterocyte migration and proliferation, making the host uniquely susceptible to further injury and finally to bacterial translocation [12].

### 2.2. Innate Immunity: The Role of Toll-Like Receptor

**Figure 2:**
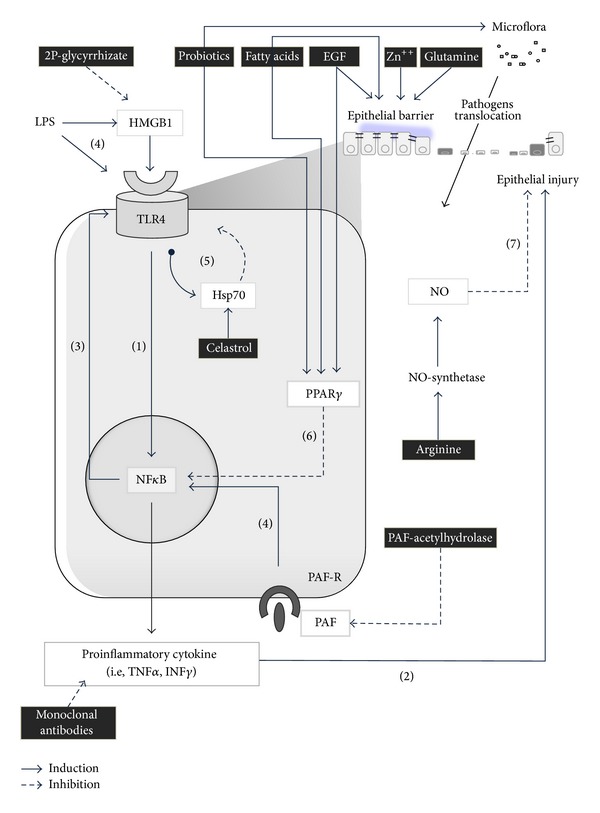
Possible therapeutic interventions on molecular mechanism inducing necrotizing enterocolitis. (1) TLR4 induces proinflammatory cytokine via the NF*κ*B pathway. (2) Proinflammatory cytokine promotes epithelial injury. (3) NF*κ*B signal increases expression of TLR4. (4) TLR signal may be induced directly by bacterial products (i.e., LPS), HMGB1, and via NF*κ*B by PAF activated receptor (PAF-R). (5) TRL4 is autoregulated by hsp70 proteins. (6) PPAR, blocking NF*κ*B pathway, is a potent inhibitor of TRL4 signal. (7) Nitric oxide (NO), produced by NO-synthetase, protects epithelium from injury. Evidenced in black-block we have reported molecules that may have potential therapeutic role in necrotizing enterocolitis, interfering with TRL4 signaling (i.e., inhibition of HMGB1 by 2-P-glycyrrhizate; induction of PPAR by probiotics, fatty acids, and EGF; PAF hydrolysis by PAF-acetylhydrolase; induction of Hsp70 by Celastrol; blocking proinflammatory cytokine by monoclonal antibodies), or maintaining epithelial barrier integrity (i.e., fatty acids, EGF, zinc, and glutamine) or protecting against epithelial injury (i.e., NO, probiotics). Please note that this is a simplified cartoon; thus reported molecules may play many other functions at mucosal and systemic level that may interfere with the development of necrotizing enterocolitis (see the text). Direct effects of TLR4 on epithelium are reported in Figure 1.

Innate immunity structured components located on the epithelial surface, which play a major role in tissue repair, are the toll-like receptors (TLRs). Among the known human TLRs, type 4 seems to have a crucial role in NEC development [12–14]. TLR4 may be activated by bacterial (i.e., lipopolysaccharides) or by other innate immunity components (i.e., high-mobility group box 1) [15]. The activation of TLR4 inhibits enterocyte migration and leads to enterocyte apoptosis in mice model, via nuclear factor kappa light chain enhancer of activated B cells (NF*κ*B) pathway activation, whereas the inhibition of TLR4 signaling in the intestinal epithelium prevents NEC development and attenuates the degree of enterocyte apoptosis in mice model and cell cultures [12–14]. Developing fetuses express elevated levels of TLR4 until the end of the gestation. This overexpression could be due to the role of TLR4 in regulating proliferation and differentiation of the intestinal epithelium during embryogenetic period [16]. The persistently elevated expression of TLR4 during intrauterine life does not increase the risk of NEC for the fetus, probably because it lives in a sterile or quasi-sterile environment. At the end of gestation, the neonate expresses low levels of TLR4 and, in the presence of a normal intestinal microflora, the signal remains inactivated. By contrast, the expression of TRL4 in preterm babies is very high, and when the premature intestine is colonized by pathologic microflora, TLR4 signal could be overactivated, leading to decreased ability to repair epithelium after injury. The final effect of this TLR4-mediated response is gut barrier failure, bacterial translocation, intestinal inflammation, and finally activation of systemic inflammatory response [12]. However, the observation that most premature infants do not develop NEC, despite the seemingly tonic activation of TLR4, suggests that TLR4 signal is somehow curtailed within the newborn intestinal epithelium, thus limiting the propensity to NEC development. It seems, therefore, that the consequences of exaggerated TLR4 signal in premature neonates can be confined by counterregulatory mechanisms that limit the consequences of TLR4 activation. These mechanisms include intra- and extracellular factors and probably may be influenced by microflora composition (Figure 2).

Heat shock proteins, of which Hsp70 is a predominant member, are a family of intracellular proteins activated by a variety of stressors, which contribute to the delivery of target proteins to the ubiquitin-proteosome system for degradation through cochaperone molecules, namely, carboxyl terminus of Hsp70-interacting protein (CHIP) [17]. Hsp70 has a protective role in the intestine limiting TLR4 signal in enterocytes. Hsp70 promotes CHIP-mediated ubiquitination and consequent degradation of TLR4 [18]. TLR4 activation itself significantly increased Hsp70 expression in enterocytes, which provides a mechanism of autoinhibition of TLR4 signal (Figure 2). Reduced activity of Hsp70 or hyperactivation of TLR disrupts this balance and induces NEC. On the contrary, upregulation of Hsp70 leads to a reduction in TLR4 signal (Figure 2) [18]. Interestingly, an extracellular factor possibly affecting TLR4 signal is the epidermal growth factor (EGF). The fetus continuously swallows amniotic fluid that limits the amplification of TLR4 signaling in the fetal intestinal mucosa and in cultured enterocytes exposed to bacterial products [19], thus markedly reducing the degree of proinflammatory cytokine release. Amniotic fluid is extremely rich in EGF. This extracellular factor inhibits TLR4 signaling via peroxisome proliferator-activated receptor gamma (PPAR*γ*) and NF*κ*B pathway (Figure 2) [19]. Other important factors, at least in part related to TLR signaling, have been recently explored in the pathogenesis of NEC (Figure 2). TLR4 signaling cans upregulate platelet-activating factor (PAF) expression thus increasing risk of injury in experimental models of NEC [20, 21]. Accumulation of ileal bile acids causes significant injury in the small intestine and acts in concert with TLR4 pathway [22]. Intestinal integrity restitution requires intercellular connectivity, mediated through small channels, namely, gap junctions, rich in connexin protein [23]. Proinflammatory cytokines (i.e., INF*γ*) cause the internalization of connexin 43, thereby impairing intercellular connectivity and reducing the extent of intestinal restitution (Figure 2) [24].

### 2.3. Gut Microbiota

Nearly all the studies on NEC associate infections with the disease. However, no specific microbe has been identified as determinant etiologic factor, and, rather surprisingly, the specific mechanisms by which infections contribute to NEC remain unknown. On the other hand, many pathogens may simulate a picture of NEC in neonates (Table 1) [7, 25–44]. The use of new molecular biology techniques has provided opportunities to reexamine this unresolved problem. Recent studies have identified an abundance of Proteobacteria (including many commonly observed Gram-negative pathogens) in fecal samples of babies that will develop NEC [45–47]. Additional demonstrated findings included a loss of gut microbial diversity and depletion of enterococcal populations in the feces, before NEC development [47]. More recently, a correlation between the clinical finding of pneumatosis intestinalis and the presence of clostridial species (*Clostridium butyricum* and* Clostridium paraputrificum*) has been proposed [48]. All these data suggest that NEC may not result from a single causative species, but more likely from a currently undefined dysbiosis, that may favor TLR4 activation and pathogens translocation across the epithelium.

## 3. Promising Therapeutic Options

The mainstay in the prevention and treatment of NEC remains a correct management of fluid intake, nutrition, prevention of infections, and adequate antibiotic therapy [1–4]. On the basis of the most recent evidences in the understanding of the pathogenic mechanisms, new therapeutic approaches can be hypothesized. Recent advances in the knowledge of pathogenic mechanism suggest that future treatment may involve immunological approaches, such as pharmacologic inhibition of TLR signal, manipulation of the intestinal environment other than administration of specific nutrients. Some options have been tested in clinical trials; many others should be developed and verified in clinical setting in the next studies (Figure 2).

### 3.1. Preservation of Epithelial Barrier by Nutritional Interventions

The adoption of adequate feeding strategies and the use of specific molecules in enteral nutrition may have a positive impact on the risk of NEC. Human milk is able to promote maturation of gastrointestinal tract in preterm neonates [49–51]. The positive effects of human milk have been attributed to several factors (i.e., macrophages, lymphocytes, sIgA, lysozyme, lactoferrin, oligosaccharides, nucleotides, cytokines, growth factors, and enzymes), but a specific component to transfer in preterm formula has not been definitively identified [50].

Endothelial nitric oxide is an important regulator of vascular perfusion and is synthesized from the amino acid L-arginine. Hypoargininemia is frequently observed in preterm neonates and may predispose them to NEC. Recently, Polycarpou et al. have demonstrated that enteral L-arginine supplementation can be safely administered in VLBW neonates and appears to reduce the incidence of stage III NEC [52]. However, larger studies are needed to further evaluate the effect of L-arginine supplementation in preventing NEC in VLBW infants [53]. Enteral glutamine supplementation decreased gastrointestinal dysfunction, number of days when feeding was withheld, and serious infectious episodes [54]. Moreover, experimental studies have shown that glutamine plays an important role in maintaining the functional integrity of the gut by serving as fuel for enterocytes, stimulating mucosal cell proliferation and differentiation, improving mucus quality, and maintaining the integrity of tight junctions [54]. Improved intestinal integrity, in turn, decreases probability of bacterial translocation and of systemic spread of bacteria [55, 56]. However, so far only animal studies have provided direct evidence for this hypothesis.

A protective role of EGF has been demonstrated mainly in animal model of NEC [57, 58]. EGF contained in amniotic fluid promotes epithelial repair by inhibition of TLR4 signal (Figure 2). EGF was tested only in one small trial including 8 cases of neonates [59]; further studies in human are advocated to verify the efficacy of EGF or amniotic fluid in preventing NEC. This study demonstrates that EGF is well tolerated and produced positive and measurable remodeling trophic effects on the gastrointestinal mucosa.

Short chain fatty acids (SCFA), derived from fermentation of undigested carbohydrates by intestinal microflora, have important effects on epithelial functions and maturation and a potent anti-inflammatory power on the mucosa [60, 61]. Despite potential therapeutic utility of SCFA in neonates at risk of NEC, no studies testing the efficacy of these molecules are available at the moment.

Zinc is ubiquitous element that participates in many metabolic pathways. The use of zinc to prevent NEC is supported by evidences demonstrating the role of zinc in maintenance of epithelial barrier function and induction of adequate immune response (Figure 2) in experimental model of NEC [62]. Recent clinical trial demonstrates the efficacy of oral zinc supplementation in reducing NEC in preterm neonates when administered at high doses [62].

### 3.2. Modulation of TLR4 Signal

The pharmacological upregulation of Hsp70 within the intestinal mucosa by Celastrol, a novel cell permeable triterpenoid antioxidant, has been tested in a mice model of NEC [63, 64]. Celastrol reduces enterocyte apoptosis and attenuates severity of NEC, but studies in human are still lacking. At the same time, it is possible to hypothesize a modulation of TLR signaling throughout different ways, as indicated in Figure 2 [19, 24, 59]. Further studies are required to establish real applicability of these novel therapeutic options to prevent and treat human cases of NEC.

### 3.3. Modification of Gut Microbiota Composition

A relatively well-studied approach to manipulate gut microbiome is the administration of probiotics. Although its efficacy in the prevention and treatment of NEC has been demonstrated in some articles [65], more works are critically needed to determine well-tolerated and effective dosing strategies and to identify the long-term effects of microbial manipulation on health and development (Table 2). The extrapolation of available evidences for probiotics to preterm neonates should take into account the characteristics of the products utilized and of the population included in the examined studies. A specific strain used in a population from a developed country may not be effective in neonates from other countries, who may have different environmental and genetic conditions. Thus, debate as to whether to give probiotics systematically to preterm infants is still ongoing. The American Pediatric Surgical Association Outcomes and Clinical Trials Committee systematic review concluded in 2012 was in support of the recent Cochrane reviews regarding the use of prophylactic probiotics in preterm infants weighing less than 2500 g to reduce the incidence of NEC [66]. However, there are no high levels of evidences to recommend the routine use of probiotics in order to decrease NEC occurrence [67]. Many aspects may have influenced the different results obtained in different trials [67]. In particular, the baseline NEC rate was a major factor affecting the potential benefit of probiotic supplementation in a specific population. The effect of probiotics decreases dramatically in areas where the occurrence of NEC is low (<5% of very low birth weight infants); hence further studies are needed before a benefit in these areas can be established. The main limitation to future trials is the large sample sizes required to demonstrate the benefit of probiotics in this setting. Mihatsch et al. calculated that, with a 5% baseline incidence of NEC, at least 714 infants per group would be required to demonstrate a 50% reduction rate by probiotics (*α* = 0.05, *β* = 0.2) [67]. Despite the larger clinical trials currently underway, there are no ongoing trials targeting a sample size that large. In addition, many drugs currently used in neonatal intensive care unit may modify microbiota composition (i.e., antibiotics, probiotics) and, thus, may influence the efficacy of such intervention of microflora manipulation.

Gastric acidity inhibitors drugs, largely used in neonatology, modify intestinal microflora and increase the risk of NEC [68, 69]. Limitation of their use should be considered as immediate efficacious strategy to reduce NEC incidence.

A novel perspective to manipulate intestinal microflora is represented by fecal transplantation. This technique has proven effective in the treatment of refractory colitis by* Clostridium difficile* and in some cases of inflammatory bowel disease [70–72]. Fecal transplantation involves direct transfer of fecal material from a healthy donor to a recipient's upper or lower intestinal tract [70–72]. No data are currently available for neonates with NEC, and important limitations should be considered before to hypothesize this therapeutic option in neonatology (i.e., modalities of transfer of fecal material from donor to recipient and/or the choice of donor).

## 4. Conclusions

A better understanding of the early mechanisms at the basis of NEC development will offer new and innovative therapeutic approaches to this severe condition. To improve the clinical management and limit the complications associated with NEC, future studies should start from the central role of the epithelium, innate immunity, and microbiota. It would be essential to explore the interaction between these main pathogenetic factors in order to hypothesize new efficacious therapeutic strategies. Further well-designed trials in selected populations are advocated to verify the efficacy of the new pharmacological and nutritional approaches hypothesized in this review.

## Figures and Tables

**Table 1:** Pathogens associated with necrotizing enterocolitis in neonates.

Bacteria species	Virus	Fungi
*Escherichia coli* [25]	*Rotavirus* [26]	*Candida albicans* [27]
*Pseudomonas aeruginosa* [28]	Adenovirus [29]	*Candida glabrata* [27]
*Klebsiella* [30]	*Norovirus* [31]	*Aspergillus fumigatus* [32]
*Cronobacter sakazakii* [33]	Astrovirus [34]	
*Shigella boydii* [35]	Echovirus [36]	
Coagulase-negative *Staphylococcus* [37]	*Cytomegalovirus* [7]	
*Clostridium* spp. [38]	Coxsackie virus [39]	
*Campylobacter* [40]	*Torovirus* [41]	
*Enterobacter cloacae* [42]	*Coronavirus* [43]	
*Salmonella* [44]		

**Table 2:** Potential benefits and demonstrated limits of probiotics.

Rational for the use of probiotics	Probiotics use in the clinical practice
Probiotics supplementation inhibits pathogenic colonization and produces anti-inflammatory effects	Neonatal Intensive Care Units with higher rates of NEC are more likely to observe a benefit with probiotic supplementation
Probiotics secrete lactic acid to lower local pH, which inhibits the growth of pathogenic bacteria	Multistrain probiotics may be more effective than single-strain products
Probiotics communicate directly with pathogenic bacteria modulating their gene expression in order to reduce binding proteins to host epithelial cells	Extremely low-birth-weight infants may not benefit to the extent observed in those with greater gestational age or body birth weight
Probiotics stimulate production of secretory immunoglobulins and positively influence immunity response	Although reports of probiotic-related sepsis are limited, caution should be used when considering probiotic supplementation in infants at greatest risk for an impaired mucosal barrier
	Policies regarding storage, preparation, distribution, administration, and documentation of probiotics to ensure patient safety should be adopted
